# Adenovirus-mediated sphingomyelin synthase 2 increases atherosclerotic lesions in ApoE KO mice

**DOI:** 10.1186/1476-511X-10-7

**Published:** 2011-01-17

**Authors:** Xiaogang Wang, Jibin Dong, Yarui Zhao, Yue Li, Manping Wu

**Affiliations:** 1 School of Pharmacy, Fudan University, Shanghai, China

## Abstract

**Background:**

Sphingomyelin synthase 2 (SMS2) contributes to de novo sphingomyelin (SM) biosynthesis. Its activity is related to SM levels in the plasma and the cell membrane. In this study, we investigated the possibility of a direct relationship between SMS and atherosclerosis.

**Methods:**

The Adenovirus containing SMS2 gene was given into 10-week ApoE KO C57BL/6J mice by femoral intravenous injection. In the control group, the Adenovirus containing GFP was given. To confirm this model, we took both mRNA level examination (RT-PCR) and protein level examination (SMS activity assay).

**Result:**

We generated recombinant adenovirus vectors containing either human SMS2 cDNA (AdV-SMS2) or GFP cDNA (AdV-GFP). On day six after intravenous infusion of 2 × 10^11 ^particle numbers into ten-week-old apoE KO mice, AdV-SMS2 treatment significantly increased liver SMS2 mRNA levels and SMS activity (by 2.7-fold, 2.3-fold, p < 0.001, respectively), compared to AdV-GFP treated mice. Moreover, plasma total cholesterol (TC), low-density lipoprotein cholesterol (LDL-C), triglyceride (TG), and sphingomyelin (SM) levels were significantly increased by 39% (p < 0.05), 42% (p < 0.05), 68% (p < 0.001), and 45% (p < 0.05), respectively. Plasma high-density lipoprotein cholesterol (HDL-C), phosphatidylcholine (PC), and PC/SM ratio were decreased by 42% (p < 0.05), 18% (p < 0.05), and 45% (p < 0.05), respectively. On day 30, the atherosclerotic lesions on the aortic arch of AdV-SMS2 treated mice were increased, and the lesion areas on the whole aorta and in the aortic root were significantly increased (p < 0.001). Furthermore, the collagen content in the aorta root was significantly decreased (p < 0.01).

**Conclusions:**

Our results present direct morphological evidence for the pro-atherogenic capabilities of SMS2. SMS2 could be a potential target for treating atherosclerosis.

## Introduction

Sphingolipids are involved in many important biological functions, including cell membrane formation, signal transduction, and lipid metabolism, which are related to the development of atherosclerosis [1]. Sphingomyelin (SM) is one of the major phospholipids. Plasma membrane SM and the ratio of SM/PC are independent risk factors for coronary heart disease [2]; furthermore, these lipids accumulate in atheromas both in humans and animal models [3]. Previous studies have shown that LDL extracted from human atherosclerotic lesions is rich in SM [4] and that plasma SM levels in apoE KO mice are four-fold higher than those in wild-type mice [5], which may partly explain the increase in atherosclerosis in these mice. SM levels were increased five-fold in VLDL from hypercholesterolemic rabbits [6]. As an inhibitor of SPT (the first key enzyme in the SM biosynthetic pathway), myriocin administration dramatically decreased plasma SM levels and atherosclerotic lesions in apoE KO mice [1,7]. These data suggest that elevated plasma SM levels could promote atherogenesis. Sphingomyelin synthase (SMS) is the last enzyme in SM biosynthesis and has two isoforms, SMS1 and SMS2. Previous work in our lab has shown that overexpression of SMS1 and SMS2 increases lipoprotein atherogenic potential in mice [8] and promotes cell apoptosis [9]. Atherogenesis is initiated by the interaction of atherogenic lipoproteins with the arterial wall [10]. Many processes have been implicated in early atherogenesis. Among them, lipoprotein retention and aggregation are key steps [11,12]. In this study, we evaluated the role of SMS2 overexpression in atherogenesis. Our results provide the first direct morphological evidence that elevated SMS activity promotes atherosclerosis in mice.

## Methods

### Mice and diets

ApoE KO mice with a C57BL/6J background were purchased from Peking University. They were divided into four groups: groups 1 and 2: AdV-SMS2 treated and groups 3 and 4: AdV-GFP treated. An appropriate aliquot of purified recombinant AdV-SMS2 or AdV-GFP (2 × 10^11 ^particle numbers) was infused into the femoral vein of 10-week-old apoE KO mice on day 0 of the study. These animals were fed a western-type diet (0.15% cholesterol, 20% saturated fat). Six days later, groups 1 and 3 were sacrificed, and liver and blood samples were collected. On day 30, groups 2 and 4 were sacrificed, and the aorta arch, aorta root, and whole aorta were isolated for analysis.

### Recombinant adenovirus generation

A pShuttle-AdEasy system was used to create adenovirus vectors (Stratagene). We generated pShuttle-CMV-SMS2 by inserting full-length SMS2 cDNAs into the SalI/NotI and NotI/EcoRV sites of pShuttle-CMV. Then, pShuttle-CMV-SMS2 was linearized by PmeI digestion and recombined with pAdEasy-1 in BJ5183 cells. Correct recombinants were selected and retransformed into Escherichia coli (DH-5α). Purified recombinant plasmids were linearized by PacI digestion and transfected into HEK-293 cells to create adenoviruses. Recombinant viruses were propagated in HEK-293 cells, purified, and titered by standard methods, as described elsewhere [8]. The corresponding viruses were named AdV-SMS2. The E1 genes (nucleotides 342-3,534) and the E3 genes (nucleotides 28,138-30,818) were deleted in those virus vectors. Recombinant adenovirus containing the GFP gene (i.e., AdV-GFP) was purchased from Viraquest Inc.

### SMS2 mRNA and activity assay

Total RNA was isolated from livers with Trizol (Invitrogen). SMS2 mRNA levels were measured by RT-PCR. Briefly, 10 μg of total RNA was reverse-transcribed with random primers and murine leukemia virus reverse transcriptase (30°C for 10 min followed by 42°C for 120 min). They were amplified by PCR with 50 nM of primers using the following cycling program: initial step of 94°C for 4 min; 21 cycles of 94°C for 30 s, 55°C for 30 s, and 72°C for 30 s; and a final step of 72°C for 8 min. The primers used for mouse SMS2 RT-PCR were as follows:

forward: 5'-CAAAACTTGAAGGTCACTTGGA-3'

reverse: 5'-GGTGGGGCTTGTGTAAGTGT-3'

The forward and reverse primer sequences for 18S rRNA (as an internal control) were 5'-AGTCCCTGCCCTTTGTACACA-3' and 5'-GATCCGAGGGCCTCACTAAAC-3', respectively. The PCR products were visualized by electrophoresis on 1.2% agarose gels, and the intensity of each band was measured by Image-Pro Plus version 4.5 software (Media Cybernetics. Inc.).

SMS activity was measured as described previously [13]. Briefly, the liver was homogenized in a buffer containing 50 mM Tris-HCl, 1 mM EDTA, 5% sucrose, and protease inhibitors. The homogenate was centrifuged at 5,000 rpm for 10 min, and the supernatant was used to measure SMS activity. The reaction system contained 10 mM HEPES (pH 7.4), 3 mM MnCl_2_, C6-nitrobenzoxadiazole-ceramide (60 mg/600 ml), and phosphatidylcholine (2 mg/600 ml). The mixture was incubated at 37°C for 2 h. Lipids were extracted in chloroform-methanol (2:1), dried under N_2 _gas, and separated by thin layer chromatography (TLC). The plate was scanned with a Phosphor imager, and the intensity of each band was measured by Image-Pro Plus version 4.5 software (Media Cybernetics, Inc.) [8].

### Plasma lipid measurement

Fasting blood was collected. Plasma TC, HDL-C, LDL-C, and TG levels were determined enzymatically by kits (Shanghai Rongsheng Biotech Co., Ltd China). Plasma SM and PC levels were measured as reported previously [8].

### Mouse Atherosclerotic Lesion Measurement

The aorta was dissected and the arch photographed, as previously reported [1]. An aortic lesion en face assay was performed as previously described [1]. For histomorphological analysis, lesional sections were stained with Oil Red O. We also performed aortic root analysis. The heart was fixed in 4% formaldehyde and embedded in paraffin. The aortic root was sectioned into 10 μm-thick slices and then stained with hematoxylin and eosin. Six sections from each aortic root, 30 μm apart from each other, were collected. Collagen analysis was performed with Masson's trichrome stain. Images were viewed and captured with a Canon microscope and treated by Image-Pro Plus 6.0.

### Statistical analysis

Differences between groups were tested by Student's t-test. Data are presented as means ± SD. P < 0.05 was considered significant.

## Results

**Figure 1:**
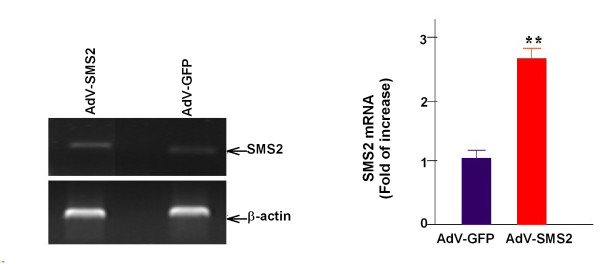
**AdV-SMS2 administration increases liver SMS2 mRNA levels**. Hepatic SMS2 mRNA levels were measured by RT-PCR. Values shown are mean ± SD, n = 6, **p < 0.001, compared to AdV-GFP.

**Figure 2:**
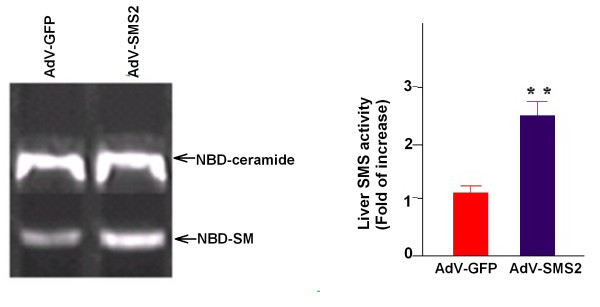
**AdV-SMS2 administration increases total SMS activity in the liver**. Values shown are mean ± SD, n = 6, **p < 0.001, compared to AdV-GFP.

To investigate the impact of elevated SMS2 activity on atherogenesis in vivo, we used AdV-SMS2 to express human SMS2 in apoE KO mice. AdV-GFP was used as a control. We then fed these animals a western-type diet. On day six after AdV-SMS2 treatment, SMS2 mRNA levels were significantly increased in the liver (2.7-fold, p < 0.001) compared to AdV-GFP treated mice (Figure 1). Moreover, AdV-SMS2 administration caused a 2.3-fold increase (p < 0.001) in liver SMS activity (Figure 2).

We next measured plasma lipid levels. As indicated in Additional file 1: Table S1, plasma lipid analysis (AdV-SMS2 versus AdV-GFP) showed a significant increase of plasma TC, LDL-C, TG and SM by 39% (p < 0.05), 42% (p < 0.05), 68% (p < 0.001), and 45% (p < 0.05), respectively and a significant reduction of plasma HDL-C, PC and PC/SM ratio by 42% (p < 0.05), 18% (p < 0.05), and 45% (p < 0.05), respectively.

**Figure 3:**
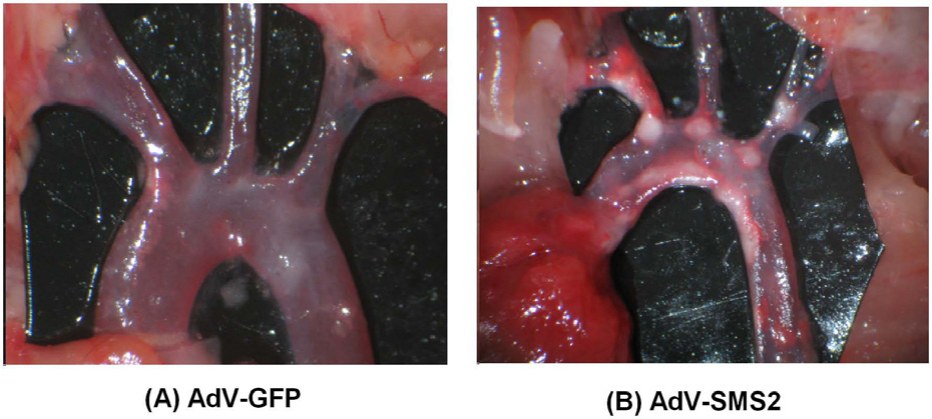
**AdV-SMS2 administration increases atherosclerotic lesions in the aortic arch. (Photograph of aortic arch)**. Panel A. Control group (AdV-GFP); Panel B. AdV-SMS2 group.

**Figure 4:**
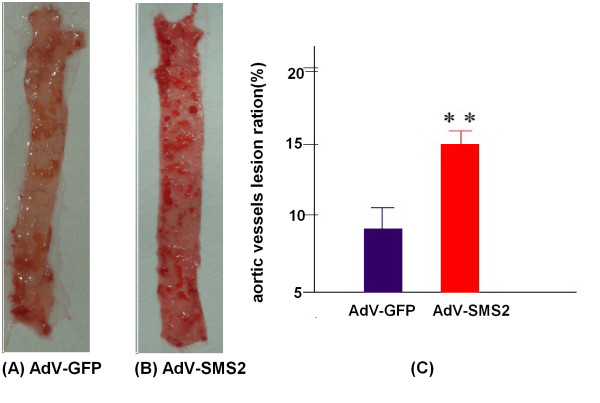
**AdV-SMS2 administration increases atherosclerotic lesions in the entire aorta. (Photograph of the entire aorta after Oil Red O staining.)**. Panel A. AdV-GFP group; Panel B. AdV-SMS2 group. Panel C. Quantification of the lesion areas. Values shown are mean ± SD (n = 7),** p < 0.001, compared to AdV-GFP.

To evaluate the impact of SMS2 overexpression on atherogenesis in apoE KO mice, we dissected the mice aortas and photographed them on day 30 after AdV-SMS2 injection. We also measured proximal and whole aortic lesion areas and found an increase of lesion area in aortic arches (Figure 3) and the whole aorta compared with controls (p < 0.001, Figure 4).

**Figure 5:**
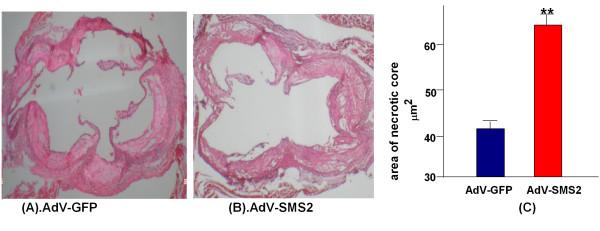
**AdV-SMS2 administration increases atherosclerotic lesions in the aorta root. (Photograph of the aorta root after H&E staining.)**. Panel A. AdV-GFP group. Panel B. AdV-SMS2 group. Panel C. Quantification of the lesion area. Values shown are mean ± SD, n = 5, **p < 0.001, compared to AdV-GFP.

**Figure 6:**
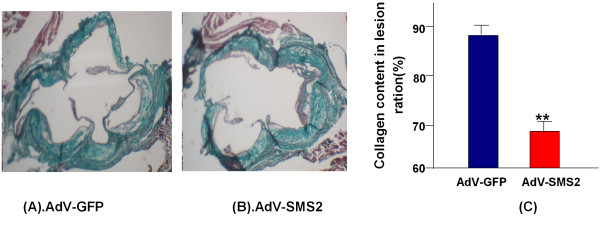
**Collage staining of lesions in the aorta root**. Panel A. AdV-GFP group. Panel B. AdV-SMS2 group. Panel C. Quantification of collagen. Values shown are mean ± SD, n = 5, **p < 0.01, compared to AdV-GFP.

We further investigated the impact of SMS2 overexpression on lesion size and collagen content in the aortic root. As shown in Figures 5 and 6, the lesion area in the aortic root was significantly increased (p < 0.001), and collage content significantly decreased (p < 0.01) compared with controls, suggesting that SMS2 overexpression not only increases lesion size but also decreases blood vessel remodeling.

## Discussion

In this study, we demonstrated that AdV-mediated SMS2 overexpression in apoE deficient background mice caused the following: 1) a significant increase in hepatic SMS2 mRNA and SMS activity levels; 2) a significant increase in plasma SM, TC, TG, and LDL-C levels and a decrease in plasma HDL-C and PC levels and in the ratio of PC/SM; and 3) an increase in atherosclerotic lesions.

SM is an amphipathic phospholipid located in the surface monolayer of all classes of plasma lipoproteins (LDL/VLDL, 70-75%; HDL, 25-30%) [14]. Human plasma SM levels are an independent risk factor for coronary heart disease [11,15]. LDL-SM retained in atherosclerotic lesions is hydrolyzed by an arterial wall sphingomyelinase, which promotes its aggregation by converting SM to ceramide [16]. We believe that reducing plasma SM levels could be an effective way to prevent atherogenesis. It has been reported that a deficiency in SMS2 causes lower plasma SM levels and less atherogenic lipoprotein aggregation mediated by sphingomyelinase [17]. Our previous study reported that SM-enriched atherogenic lipoprotein particles from AdV-SMS2 treated mice or liver-specific SMS2 transgenic mice have a stronger potential for aggregation after sphingomyelinase treatment [8]. The present study is the first to indicate that elevated SM levels caused by increased SMS activity promote atherogenesis in a mouse model.

Collagen is synthesized by vascular smooth muscle cells and plays an important role during blood vessel remodeling [18]. During atherogenesis, many inflammatory factors and cytokine are overexpressed, leading to vascular smooth muscle cell apoptosis and decreased collagen synthesis. There is a negative relationship between collagen content and atherosclerosis [19]. The present study shows that SMS overexpression not only increases atherosclerotic lesions but also decreases collagen content, implying a decrease in the ability for blood vessel remodeling.

The interaction of SM and cholesterol drives the formation of plasma membrane rafts [20,21]. Adenovirus-mediated SMS2 expression might alter lipid raft structure, which is supported by the fact that AdV-mediated SMS1 and SMS2 overexpression can upregulate scavenger receptor B-I (SR-BI) [8], which is closely associated with lipid rafts on the plasma membrane [21-23]. SR-BI can mediate the selective uptake of cholesteryl esters from both LDL and HDL particles, but these processes are affected differently by the integrity of lipid rafts on hepatocyte membranes [24]. This effect could explain why there is a reduction of HDL-C (Additional file 1: Table S1).

## Conclusion

In summary, our results provide the first direct morphological evidence that SMS2 overexpression can promote atherosclerosis in a mouse model. Thus, SMS2 could be a new target for atherosclerosis treatment.

## Competing interests

The authors declare that they have no competing interests.

## Authors' contributions

XGW participated in the investigation, analysis and interpretation of data, drafting the manuscript. JBD contributed to conception and design, experimental protocol, revised the manuscript. YRZ participated in the investigation, acquisition of data. YL had taken responsibilities in the adenovirus preparation and participated into the animal experiment. MPW designed the study and revised the manuscript, and given final approval of the version to be published. All authors have read and approved the final manuscript.

## Supplementary Material

Additional file 1 **Table S1**. Plasma lipid levels in mice overexpressing SMS2 and in control mice. Click here for file
