# Supplementary figures and images for: Adenovirus-mediated sphingomyelin synthase 2 increases atherosclerotic lesions in ApoE KO mice
Source: Lipids Health Dis. 2011 Jan 17;10:7. doi: 10.1186/1476-511X-10-7 (PMC3032723; doi:10.1186/1476-511X-10-7)

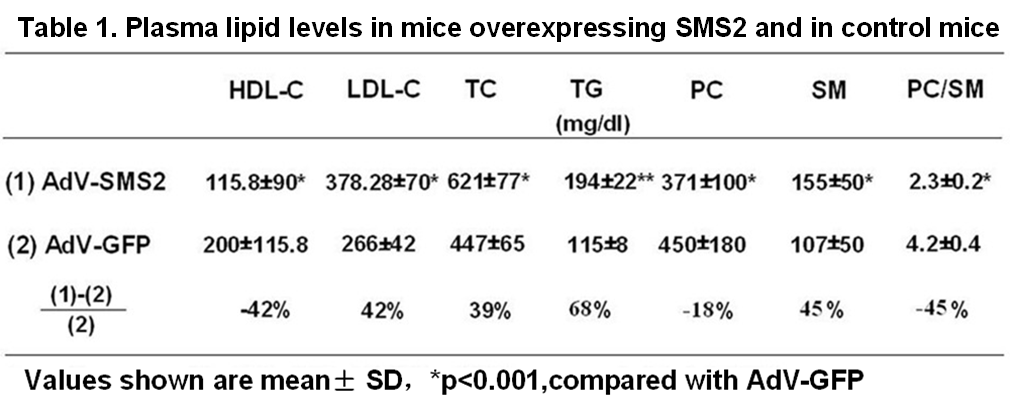

Supplement: Additional file 1 — Table S1. Plasma lipid levels in mice overexpressing SMS2 and in control mice. [file 1476-511X-10-7-S1.TIFF]
